# The use of technology in tracking soccer players’ health performance: a scoping review

**DOI:** 10.1186/s12911-020-01156-4

**Published:** 2020-08-11

**Authors:** Jassim Almulla, Abdulrahman Takiddin, Mowafa Househ

**Affiliations:** 1 College of Science and Engineering, Hamad Bin Khalifa University, Doha, Qatar; 2 Qatar Football Association, Doha, Qatar; 3 Texas A&M University at Qatar, Doha, Qatar

**Keywords:** Soccer, Player, Health, Performance, Technology

## Abstract

**Background:**

Quantifying soccer players’ performance using different types of technologies helps coaches in making tactical decisions and maintaining players’ health. Little is known about the relation between the performance measuring technologies and the metrics they measure. The aim of this study is to identify and group the different types of technologies that are used to track the health-related performance metrics of soccer players.

**Methods:**

We conducted a systematic search for articles using IEEE Xplore, PubMed, ACM DL, and papers from the Sports Medicine Journal. The papers were screened and extracted by two reviewers. The included papers had to fall under several criteria, including being about soccer, measuring health-related performance, and using technology to measure players’ performance. A total of 1,113 papers were reviewed and 1,069 papers were excluded through the selection process.

**Results:**

We reviewed 44 papers and grouped them based on the technology used and health-related metrics tracked. In terms of technology, we categorized the used technologies into wearable technologies (N=27/44) and in-field technologies (N=14/44). We categorized the tracked health-related metrics into physiological metrics (N=16/44) and physical metrics (N=44/44). We found out that wearable technologies are mainly used to track physical metrics (N=27/27) and are also used to track physiological metrics (N=14/27). In-field technologies are only used to track physical metrics (N=24/24).

**Conclusion:**

Understanding how technology is related to players’ performance and how it is used leads to an improvement in the monitoring process and performance outcomes of the players.

## Background

As soccer is gaining popularity around the world, soccer organizations and researchers are attempting to improve the overall performance of the players, maintain their health, and win more matches [1]. The performance of the players is being tracked in official matches as part of local or international competitions [2], and in to unofficial matches such as team’s training sessions, training matches among team players, and friendly matches with other teams [3, 4]. Official matches tend to be more intense than unofficial matches due to the pressure to win these matches.

Soccer coaches are interested in technical and health-related performance metrics [5] as both are essential when it comes to quantifying the overall performance of the players [6, 7]. Technical performance metrics include players’ activities during the match, such as the number of successful passes, duration of ball possession, number of passes among players, and number of shots the player attempts to score a goal [1, 8]. The health-related performance metrics include the total distance covered by players in moderate, high, and very high speeds [5].

To monitor the players’ performance, soccer organizations and researchers are using different types of tracking technologies that are capable of tracking different metrics of the players. These technologies replace the manual process where specialized observers review taped videos of the matches and code players’ activity patterns [3]. These technologies help reduce the time needed to collect data about the players during matches and assist coaches in gathering more data about different aspects of the players.

The aim of this study is to identify the different technologies used to track performance metrics of soccer players in recent literature. The study also identifies and categorizes the different health-related performance of soccer players. Identifying and linking the various technologies to health-related metrics help coaches and health practitioners in the sports field to focus on many aspects of the player to maintain players’ health conditions, which leads to enhance the overall performance of the players.

## Method

### Search strategy

We conducted a systematic search and included studies up until the 31st of March, 2019. We identified the articles from IEEE Xplore, PubMed, and ACM DL. In order to have more specialized journals in the sports field, we searched within SJR’s (SCImago Journal Rank) list of highly ranked journals under the “Sports Science” category and chose the Sports Medicine Journal, which is one of the most highly ranked international journals. Then, we identified the papers using the “Springer Link” database search engine.

We searched IEEE Xplore, ACM DL, and Springer Link databases using the combination of the following keywords: (1) “soccer”, “football”; (2) “technology”, “system”; (3) “athlete”, “player”; (4) “performance”. We connected the terms with the “OR” term within each of the four combination groups. We combined the four combination groups using the “AND” term. The exact phrasing of the search keywords is:


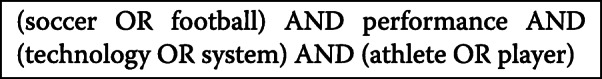


In the Pubmed database, we used the “MeSH” (Medical Subject Headings) approach, which is the national library of medicine’s controlled vocabulary thesaurus that is used for indexing and organizing articles. Each article is associated with MeSH terms that describe the content of the citation. Our MeSH search term included “Soccer” and “Athletic Performance” as two mesh phrases joined by the “AND” keyword. Then that, we separated the keywords “technology” and “system” by the “OR” keyword. The exact phrasing of the search keywords is:


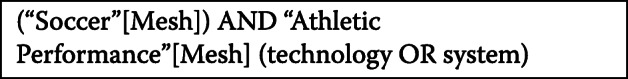


### Selection criteria

**Fig. 1:**
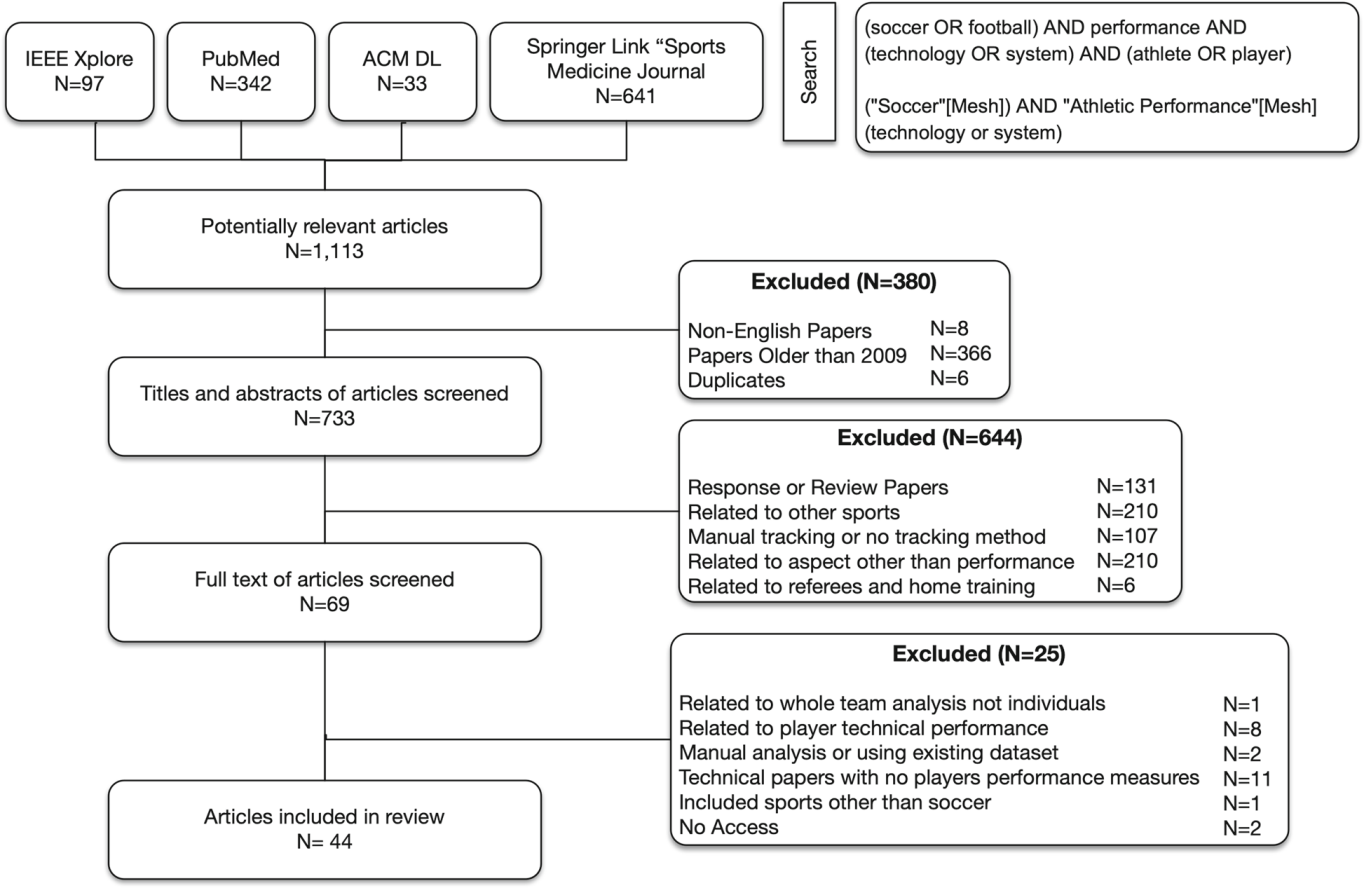
PRISMA Diagram

The selection criteria was based on four main requirements. First, the study had to be about soccer only. We used the keyword “football” since it is used to refer to soccer in some countries, and excluded any study that discussed American football, rugby, or futsal (indoor soccer). Second, the study had to focus on players’ performance. As such, we excluded studies that assessed the performance of a particular technology. Third, the study had to be about monitoring players’ performance through technology. Thus, we excluded studies that monitored players’ performance through manual monitoring techniques. Finally, the paper had to be published within the last ten years (i.e. 2009-2019). To visualize our search process, we used the Preferred Reporting Items for Systematic Reviews and Meta-Analyses (PRISMA), as illustrated in Fig. 1.

After completing the basic search, we had a total of (N=1113) papers. Then, we started excluding papers in three phases. In the first phase, we excluded papers (N=380) due to the following reasons: non-English papers (N=8), papers published before 2009 (N=366), and duplicate papers (N=6).

In the second phase, we, the two assessors (JM an AT), independently assessed a total of (N=733) papers using the title and abstract of the article. We excluded a number of papers (N=644) based on the following exclusion criteria. We excluded: (1) response or review papers (N=131); (2) papers related to sports other than soccer, such as American football, rugby, and futsal (N=210); (3) papers that used manual performance tracking approaches or papers that did not specify the used performance tracking method (N=107); (4) papers that were not related to player’s performance, such as player’s injury, strategy, or recovery (N=210); and (5) papers that were not related professional players, such as referees and home training (N=6).

In the last phase, we included a total of 69 papers (N=69) for full-text review. After reviewing the full text, we excluded some papers (N=25) based on the following criteria. We excluded: (1) studies that analyzed a whole team, but not individual players within the team (N=1); (2) papers related to players’ technical performance, such as the number of passes (N=8); (3) studies that used manual performance analysis or used existing datasets without involving technology in the analysis (N=2); (4) technical papers that did not discuss players’ performance metrics (N=11); (5) studies that did not focus on soccer only but rather discussed other sports (N=1); and (6) papers that the assessors could not access (N=2).

## Results

### Study characteristics

**Fig. 2:**
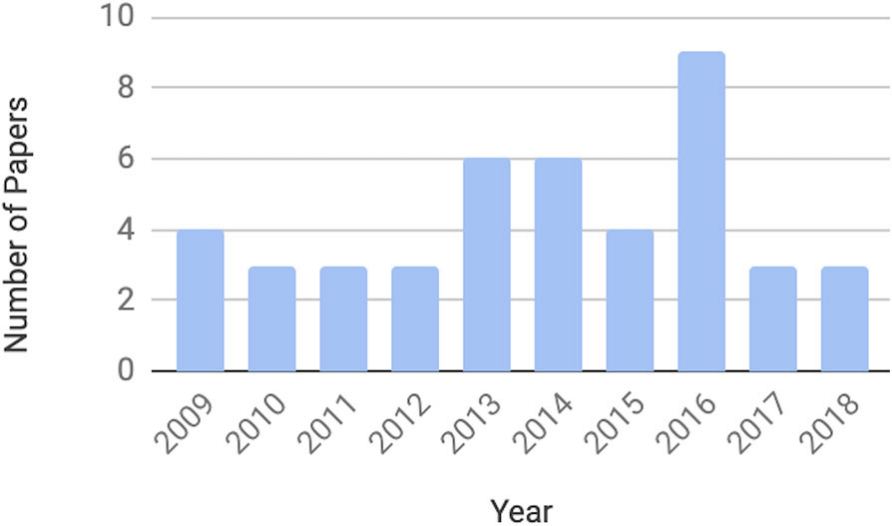
Number of published papers per year

**Fig. 3:**
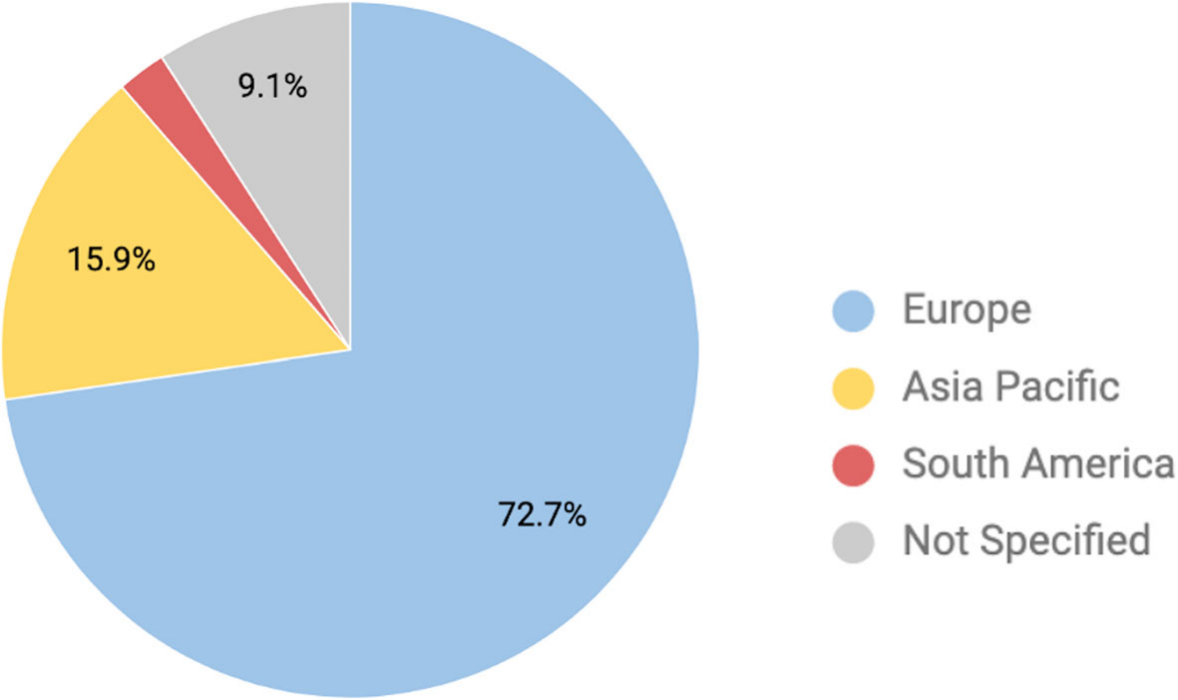
Study Location per region

Figure 2 shows the number of papers published per year since 2009. We observed that the number of papers was increasing until 2016 (N=9/44). Then, the number of published papers dropped in the following two years. We also analyzed the location of the study, as shown in Fig. 3. Around 72% (N=32/44) of the studies were conducted in Europe, 16% (N=7/44) in Asia-Pacific region, and 2% (N=1/44) in South America. The rest of the papers did not specify the location of the study (N=4/44).

Table 1 summarizes the sample size, gender, age group, and the study duration of the conducted studies in the papers. The number of participants in the studies varies from 6 to more than 26,000 players. More than 86% (N=38/44) of the papers experimented with male players while only one paper (N=1/44) studied female players. The rest of the papers (N=5/44) did not specify the gender. The number of matches in the studies ranged from 1 match to 460 matches.

**Table 1:** Summary findings

Reference	Sample Size	Gender	Age Group	Duration	Matches
(Ric et al. 2017) [1]	19	M	Senior	1 D	1
(Wehbe et al. 2014) [2]	19	M	Senior	1 S	8
(Malone et al, 2018) [5]	48	M	Senior	1 S	460
(Scott et al. 2013) [6]	15	M	Senior	1 S	29
(Moalla et al. 2018) [8]	17	✗	Senior	2 S	52
(Akenhead et al. 2016) [44]	33	M	Senior	39 W	195
(Castagna et al. 2017) [9]	1,200	M	Senior	1 S	60
(Torreño et al. 2016) [10]	26	M	Senior	1 S	✗
(Rossi et al. 2016) [11]	26	M	Senior	1 S	80
(Bradley et al. 2014) [12]	810	M	Senior	1 S	20
(Di Mascio and Bradley 2013) [13]	100	M	Senior	1 S	20
(Carling et al. 2016) [14]	12	M	Senior	1 S	31
(Ingebrigtsen et al. 2015) [15]	15	M	Senior	1 S	15
(Varley and Aughey 2013) [16]	29	M	Senior	1 S	34
(Vigne et al. 2010) [17]	25	M	Senior	1 S	30
(Andrzejewski et al. 2012) [18]	31	M	Senior	1 S	4
(Castellano et al. 2011) [19]	434	M	Senior	1 S	✗
(Dalen et al. 2016) [20]	45	M	Senior	3 S	✗
(Bradley et al. 2013) [21]	711	M	Senior	3 S	✗
(Di Salvo et al. 2009) [22]	563	M	Senior	3 S	✗
(Di Salvo et al. 2012) [23]	26,449	M	Senior	4 S	✗
(Bradley et al. 2016) [24]	✗	M	Senior	7 S	1
(Bush et al. 2015) [25]	1,036	M	Senior	7 S	✗
(Rampinini et al. 2009) [26]	186	M	Senior	✗	416
(Randers et al. 2010) [3]	20	✗	Youth	1 D	1
(Stevens T et al. 2014) [4]	12	M	Youth	✗	✗
(Russell et al. 2016) [27]	11	M	Youth	1 S	19
(Casamichana et al. 2012) [28]	27	M	Youth	2 S	✗
(Hodgson et al. 2014) [29]	8	M	Youth	4 S	3
(Mugglestone et al. 2013) [30]	20	M	Youth	✗	50
(Bendiksen et al. 2013) [31]	11	F	Youth	✗	✗
(Varley et al. 2017) [32]	6	✗	Youth	✗	✗
(Harley et al. 2011) [33]	6	M	Youth	✗	✗
(Coutinho et al. 2018) [7]	90	M	Young	✗	✗
(Fernandes-da-Silva et al. 2016) [34]	33	M	Young	3 W	9
(Hill-Haas et al. 2009) [35]	16	M	Young	9 W	✗
(Abade et al. 2014) [36]	151	M	Young	9 W	38
(Abade et al. 2014) [37]	151	M	Young	1 S	38
(Goto et al. 2015) [38]	34	✗	Young	2 S	✗
(Buchheit et al. 2010) [39]	77	M	Young	8 S	✗
(Castagna et al. 2009) [40]	21	M	Young	✗	✗
(Goto et al. 2015) [41]	81	M	Young	✗	✗
(Buchheit et al. 2011) [42]	104	✗	Young	✗	66
(Nagahara et al. 2016) [43]	20	M	✗	✗	2

Legend: ✗ = Information Not Available, M = Male, F = Female, D = Day, W = Week, S = Season

The age of the players is addressed in the papers using 2 representations; standard age range in years and the soccer notation, such as senior (players in the first league) and U23 (under 23 years old). The age of the players in the studies ranges from 9 to 30 years old. We categorized the ages of the participating players into three main groups: (1) Senior: players above 23 years old; (2) Youth: players between 18 and 23 years old; and (3) Young: players below 18 years old. More than 54% of the papers (N=24/44) focused on senior players, around 23% of the papers studied young players (N=10/44), and around 20% of the papers studied youth players (N=9/44). One paper did not specify the age of the studied players (N=1/44).

The duration of the studies ranged from 1 day to 7 soccer seasons. Out of the 44 papers, 34 papers specified the duration of the conducted studies (N=34/44), whereas 10 papers did not specify (N=10/44). We grouped the duration of the studies into three groups: (1) one-day-study: studies conducted in one day (N=2/34); (2) weeks: studies conducted over weeks (N=5/34), which ranged from 3 to 39 weeks; and (3) seasons: studies that lasted for one or more soccer seasons (N=24/34). In the seasons group, 66% of the papers were conducted during 1 season (N=16/24), and the rest of the papers lasted between 2 and 7 seasons (N=8/24).

### Type of match

We divided the studies into two groups according to the match type used for data collection: studies that were conducted on players during official matches and studies that were conducted on players during unofficial matches. Approximately 91% (N=40/44) of the papers specified the match type. Out of the 40 studies, 40% (N=16/40) of the papers conducted their experiments with players during official matches, whereas 60% (N=24/40) experimented with players during unofficial sessions. The rest of the studies (N=4/44) did not specify the match type of the conducted experiments.

### Tracking technologies

We extracted different types of technologies used to track the players during matches. Global Positioning System (GPS) is used to measure the position of the players for each second in time [1, 9]. Local Position Measurement System (LPMS) is a system that constantly tracks the players’ body movements and acceleration [4]. Heart Rate Monitor (HRM) is a device that is used to monitor the heart rate of the players and associate it with different actions and events [10]. Accelerometer (ACC) is a motor sensor that quantifies the physical activities and physiological demands of the players; it is highly responsive to the acceleration of the body movements and records it in three dimensions [20]. Gyroscope (GS), Digital Compass (DC), and Microelectromechanical System (MEMS) are components within the GPS that are used to improve the accuracy of the readings [11, 44]. In addition to these technologies, sensors, which are devices attached to the players, are used to transfer the collected data by microwave radio channels to other sensors mounted on the soccer arena [20]. Also, the Multi-Camera System (MCS) consists of cameras mounted on the stadium’s roof and the footage is analyzed using proprietary software [12, 23].

As shown in Table 2, we divided all the performance monitoring technologies into two groups. First, **wearable technologies** include the devices that are attached to the players to monitor their performance. Approximately 68% of the papers (N=30/40) used wearable technologies to track the performance of the players. Approximately 90% of those studies (N=27/30) used GPS technology. The other wearable technologies are GS, DC, MEMS, LPMS, HRM, ACC, and sensors, which complement the GPS to get more specific and accurate data about the players. Second, **in-field technologies** include the devices that are installed in the soccer arena to monitor the players. Approximately 39% of the studies (N=24/44) used in-field technology to track the performance of the players. The in-field technology refers to the MCS. Only 9% of the studies (N=4/44) used both wearable and in-field technologies to track the performance of the players.

**Table 2:** Players’ performance tracking technologies in soccer

Category	Technology	Sources
Wearable	GPS	[1–7, 9–11, 16, 27–30, 32–39, 41–44]
	GS	[11]
	DC	[11]
	MEMS	[44]
	LPMS	[4]
	HRM	[5, 6, 10, 35–37, 40]
	ACC	[5, 6, 10, 11, 20, 28, 30, 37]
	Sensor	[15, 20, 31]
In-Field	MCS	[1–7, 10, 11, 15, 16, 28–31, 34–37, 39–41, 43, 44]

### Performance metrics

Different health-related metrics were extracted from the papers. The Heart Rate (HR) of the players is usually measured in predefined time intervals [35]. The Metabolic Power (MP) is a method that shows the soccer player’s specific activity using speed and acceleration [9]. Muscular Fatigue (MSF) is when the muscles of players are unable to generate force [7]. The Distance Covered (DC) is the quantified distance that each player covers during the match. Speed (SP) refers to the running speed of the players. Acceleration and Deceleration (AD) refer to the change of speed of soccer players during a match.

We categorized the performance metrics into two main categories, as shown in Table 3. The first category is the physiological metrics, which includes the metrics that are related to the functions of the players’ internal organs. The physiological metrics are the HR, MP, and MSF. Approximately 36% (N=16/44) of the papers studied the physiological metrics of the players, 69% (N=11/16) of those papers focused specifically on HR, four papers discussed MP (N=4/16), and two papers discussed MSF (N=2/16). The second category is the physical metrics, which includes metrics related to the physical activity of the players during the soccer match. The physical metrics are the DC, SP, and AD. All of the studied papers (N=44/44) discussed the physical performance of the players.

**Table 3:** Soccer players’ performance metrics

Category	Performance Metric	Sources
Physiological	HR	[5, 6, 10, 29, 31, 34–37, 40, 44]
	MP	[4, 9, 11, 29]
	MSF	[7, 11]
Physical	DC	[5, 6, 8–12, 20, 24–26, 29, 33, 34, 36, 40]
	SP	[1–10, 12, 13, 15–44]
	AD	[2, 4, 7, 9–11, 15, 16, 20, 27, 32, 37, 39, 44]

### Relationship between findings

Table 4 summarizes and links the studies according to the match type, technology type, and the performance measured. Out of the studies conducted during official matches (N=16/40), most of them (N=13/16) used in-field technologies only, one study (N=1/16) used wearable technologies, and the remaining (N=2/16) used both wearable and in-field technologies to measure the performance of the players. Out of the studies conducted during unofficial matches (N=24/40), around 91% of them (N=22/24) used only wearable technologies and the rest (N=2/24) used both wearable and in-field technologies as their tracking methods. In 61% of the papers (N=27/44), wearable technologies were used to track the performance metrics. Wearable technologies were used to track both physical and physiological performance. Wearable technologies were used to track players’ physical performance in all papers (N=27/27). Wearable technologies were also used in 52% of the studies (N=14/27) to track physiological performance. On the other hand, in 32% of the studies (N=14/44), in-field technologies were used to track the physical performance of the players only, whereas the physiological metrics were never tracked using in-field technologies.

**Table 4:** Technology types used in matches

Source	Match Type	Technology Type		Performance Measured	
		Wearable	In-Field	Physiological	Physical
(Castagna et al. 2017) [9]	Official	$\checkmark $	$\checkmark $	✗	$\checkmark $
(Dalen et al. 2016) [20]	Official	$\checkmark $	$\checkmark $	✗	$\checkmark $
(Moalla et al. 2018) [8]	Official	✗	$\checkmark $	✗	$\checkmark $
(Bradley et al. 2014) [12]	Official	✗	$\checkmark $	✗	$\checkmark $
(Di Mascio and Bradley 2013) [13]	Official	✗	$\checkmark $	✗	$\checkmark $
(Carling et al. 2016) [14]	Official	✗	$\checkmark $	✗	$\checkmark $
(Vigne et al. 2010) [17]	Official	✗	$\checkmark $	✗	$\checkmark $
(Andrzejewski et al. 2012) [18]	Official	✗	$\checkmark $	✗	$\checkmark $
(Castellano et al. 2011) [19]	Official	✗	$\checkmark $	✗	$\checkmark $
(Bradley et al. 2013) [21]	Official	✗	$\checkmark $	✗	$\checkmark $
(Di Salvo et al. 2009) [22]	Official	✗	$\checkmark $	✗	$\checkmark $
(Di Salvo et al. 2012) [23]	Official	✗	$\checkmark $	✗	$\checkmark $
(Bradley et al. 2016) [24]	Official	✗	$\checkmark $	✗	$\checkmark $
(Bush et al. 2015) [25]	Official	✗	$\checkmark $	✗	$\checkmark $
(Rampinini et al. 2009) [26]	Official	✗	$\checkmark $	✗	$\checkmark $
(Russell et al. 2016) [27]	Official	$\checkmark $	✗	✗	$\checkmark $
(Ric et al. 2017) [1]	Unofficial	$\checkmark $	✗	✗	$\checkmark $
(Wehbe et al. 2014) [2]	Unofficial	$\checkmark $	✗	✗	$\checkmark $
(Ingebrigtsen et al. 2015) [15]	Unofficial	$\checkmark $	✗	✗	$\checkmark $
(Varley and Aughey 2013) [16]	Unofficial	$\checkmark $	✗	✗	$\checkmark $
(Mugglestone et al. 2013) [30]	Unofficial	$\checkmark $	✗	✗	$\checkmark $
(Buchheit et al. 2010) [39]	Unofficial	$\checkmark $	✗	✗	$\checkmark $
(Goto et al. 2015) [41]	Unofficial	$\checkmark $	✗	✗	$\checkmark $
(Nagahara et al. 2016) [43]	Unofficial	$\checkmark $	✗	✗	$\checkmark $
(Malone et al, 2018) [5]	Unofficial	$\checkmark $	✗	$\checkmark $	$\checkmark $
(Scott et al. 2013) [6]	Unofficial	$\checkmark $	✗	$\checkmark $	$\checkmark $
(Coutinho et al. 2018) [7]	Unofficial	$\checkmark $	✗	$\checkmark $	$\checkmark $
(Akenhead et al. 2016) [44]	Unofficial	$\checkmark $	✗	$\checkmark $	$\checkmark $
(Torreño et al. 2016) [10]	Unofficial	$\checkmark $	✗	$\checkmark $	$\checkmark $
(Rossi et al. 2016) [11]	Unofficial	$\checkmark $	✗	$\checkmark $	$\checkmark $
(Casamichana et al. 2012) [28]	Unofficial	$\checkmark $	✗	$\checkmark $	$\checkmark $
(Hodgson et al. 2014) [29]	Unofficial	$\checkmark $	✗	$\checkmark $	$\checkmark $
(Bendiksen et al. 2013) [31]	Unofficial	$\checkmark $	✗	$\checkmark $	$\checkmark $
(Fernandes-da-Silva et al. 2016) [34]	Unofficial	$\checkmark $	✗	$\checkmark $	$\checkmark $
(Hill-Haas et al. 2009) [35]	Unofficial	$\checkmark $	✗	$\checkmark $	$\checkmark $
(Abade et al. 2014) [36]	Unofficial	$\checkmark $	✗	$\checkmark $	$\checkmark $
(Castagna et al. 2009) [40]	Unofficial	$\checkmark $	✗	$\checkmark $	$\checkmark $
(Abade et al. 2014) [37]	Unofficial	$\checkmark $	✗	$\checkmark $	$\checkmark $
(Randers et al. 2010) [3]	Unofficial	$\checkmark $	$\checkmark $	✗	$\checkmark $
(Stevens T et al. 2014) [4]	Unofficial	$\checkmark $	$\checkmark $	$\checkmark $	$\checkmark $
(Varley et al. 2017) [32]	✗	$\checkmark $	✗	✗	$\checkmark $
(Harley et al. 2011) [33]	✗	$\checkmark $	✗	✗	$\checkmark $
(Goto et al. 2015) [38]	✗	$\checkmark $	✗	✗	$\checkmark $
(Buchheit et al. 2011) [42]	✗	$\checkmark $	✗	✗	$\checkmark $

Legend: $\checkmark $ = Measured, ✗ = Not Measured

A few papers (N=4/44) used both wearable and in-field technologies to track the performance of the players. In two studies [3, 33], the authors compared the outcomes of wearable and in-field technologies. In another study [4], the wearable technologies were the main tracking method used for the study and the in-field technology was used as a gold standard, which means that it was used just to confirm the results.

## Discussion

Our paper summarized the findings in the literature to identify and group the different health-related performance metrics tracked for soccer players along with the technologies used to track these metrics in official and unofficial matches. All of the papers we reviewed conducted experiments with soccer players using different technologies to assess different performance aspects without relating the use of technology to the performance aspects. Our contribution in this paper filled this gap by linking each technology used with the performance metrics it measures. We also identified the type of match that the technology is used in. To the best of our knowledge, no similar work has been done to fill this gap.

Based on this review, there were only three papers that studied the performance of soccer players in official matches using wearable technologies. These studies were conducted in 2016 and 2017. In 2015, the International Football Association Board (IFAB) approved the use of wearable technologies in official matches [45]. Before 2015, in-field technologies were the main method to monitor the performance of the players in official matches, whereas wearable technologies were used in unofficial matches. In-field technologies are not usually used during unofficial matches due to the high cost of operation and the need for a specialized operator to run the system [3]. Also, in-field technologies require special installation in the club’s main stadium, where only some matches take place [33]. Training sessions and unofficial matches are sometimes conducted in training fields where in-field technologies are not available. For that reason, researchers and team officials rely on wearable technologies to track the performance of players during training sessions and unofficial matches as it is portable and relatively easier to operate.

In-field technologies are only used to track the physical performance of the players as MCS can automatically track the CD, SP, and AD of the players. However, studying the physiological aspects of the players has to be done through direct interaction with the player’s body, which cannot be achieved using the in-field multi-cameras system. The wearable technologies are used to track all different types of health-related performance metrics required by the researchers. There are a few papers that used both types of technologies in their studies. However, their main purpose was to compare the results of both technologies.

The physical metrics were tracked by all the papers in our study. We discovered that there are three main physical metrics that were tracked in the papers: (1) distance covered, (2) speed, and (3) acceleration and deceleration. Tracking the physical performance is crucial since it is directly related to the performance of the team during a match, which also affects the technical performance of the players and eventually winning a game. Both in-field and wearable technologies were used to track the physical performance of the players. Mainly, wearable technologies were used to track the players’ physical performance during unofficial matches, whereas in-field technologies were used to track the physical performance during official matches.

The physiological performances of the players were tracked using wearable devices only in the studied papers. We identified three main physiological metrics: (1) HR, (2) MP, and (3) MSF. All the included studies that analyzed physiological performance were on unofficial matches. We believe that more work has to be done regarding the physiological performance during official matches since players tend to have higher intensity activities compared to unofficial matches. Studying the physiological performance during official matches will allow coaches and researchers to understand and improve players’ performance during official matches accordingly.

Most of the studied papers focused on the physical performance of the players in the first place, which is due to the ease of measuring the physical performance using the different types of technologies. Also, monitoring the physical performance of the players does not require special skills to analyze and understand the readings by coaches, players, and technical staff. However, monitoring the physiological performance requires more specialized practitioners and needs specific skills to interpret the results.

### Limitation

This scoping review examined papers written in English; other languages were not included, which might have excluded some studies conducted in other parts of the world like South America. A limitation might be the gap between the time the research was done and the time the work was submitted, which will exclude published papers during that period. We conducted a systematic search in the academic databases to include all published papers in this area, but we might have missed some. Although we tried to discuss all the findings in the literature, it is impossible to detail all the findings found in the papers.

### Future work

There is a need to compare the performance of the in-field technologies with the wearable technologies to track the physical performance of the players. Also, future research should focus more on the physiological aspects of the players as it helps in understanding the players’ health status. More studies are required on female players due to the lack of studies conducted on them compared to male players. It is also important to link the monitored metrics to the health of the players.

## Conclusion

Technology has automated the process of measuring soccer players’ performance. Two types of technologies are used to monitor health-related performances. Players’ physiological performances are determined primarily using wearable technologies. The physical performance of the player is measured using both wearable and in-field technologies. Understanding the relationship between technology and performance as well as how and where it is being used helps in improving the monitoring process, which leads to improving the overall performance of the players.

## Data Availability

The datasets used and/or analysed during the current study are available from the corresponding author on reasonable request.

## Abbreviations

ACC: Accelerometer
AD: Acceleration &Deceleration
DC: Distance Covered
GPS: Global Positioning System
GS: Gyroscope
HR: Heart Rate
HRM: Heart Rate Monitor
LPMS: Local Position Measurement System
MCS: Multi-Camera System
MEMS: Microelectromechanical System
MeSH: Medical Subject Headings
MP: Metabolic Power
MSF: Muscular Fatigue
PRISMA: Preferred Reporting Items for Systematic Reviews and Meta-Analyses
